# Prenatal anxiety and obstetric decisions among pregnant women in Wuhan and Chongqing during the COVID‐19 outbreak: a cross‐sectional study

**DOI:** 10.1111/1471-0528.16381

**Published:** 2020-08-02

**Authors:** X Liu, M Chen, Y Wang, L Sun, J Zhang, Y Shi, J Wang, H Zhang, G Sun, PN Baker, X Luo, H Qi

**Affiliations:** ^1^ Department of Obstetrics The First Affiliated Hospital of Chongqing Medical University Chongqing China; ^2^ China‐Canada‐New Zealand Joint Laboratory of Maternal and Fetal Medicine Chongqing Medical University Chongqing China; ^3^ Maternal and Child Health Hospital of Hubei Province Wuhan China; ^4^ First Clinical Institute Chongqing Medical University Chongqing China; ^5^ School of Public Health and Management Chongqing Medical University Chongqing China; ^6^ MOE‐Shanghai Key Laboratory of Children's Environmental Health Xin Hua Hospital Affiliated to Shanghai Jiao Tong University School of Medicine Shanghai China; ^7^ Department of Neonatology Children's Hospital of Chongqing Medical University Chongqing China; ^8^ College of Life Sciences University of Leicester Leicester UK

**Keywords:** COVID‐19, obstetric decisions, prenatal anxiety

## Abstract

**Objectives:**

To investigate the mental status of pregnant women and to determine their obstetric decisions during the COVID‐19 outbreak.

**Design:**

Cross‐sectional study.

**Setting:**

Two cities in China––Wuhan (epicentre) and Chongqing (a less affected city).

**Population:**

A total of 1947 pregnant women.

**Methods:**

We collected demographic, pregnancy and epidemic information from our pregnant subjects, along with their attitudes towards COVID‐19 (using a self‐constructed five‐point scale). The Self‐Rating Anxiety Scale (SAS) was used to assess anxiety status. Obstetric decision‐making was also evaluated. The differences between cities in all of the above factors were compared and the factors that influenced anxiety levels were identified by multivariable analysis.

**Main outcome measures:**

Anxiety status and its influencing factors. Obstetric decision‐making.

**Results:**

Differences were observed between cities in some background characteristics and women's attitudes towards COVID‐19 in Wuhan were more extreme. More women in Wuhan felt anxious (24.5 versus 10.4%). Factors that influenced anxiety also included household income, subjective symptom and attitudes. Overall, obstetric decisions also revealed city‐based differences; these decisions mainly concerned hospital preference, time of prenatal care or delivery, mode of delivery and infant feeding.

**Conclusions:**

The outbreak aggravated prenatal anxiety and the associated factors could be targets for psychological care. In parallel, key obstetric decision‐making changed, emphasising the need for pertinent professional advice. Special support is essential for pregnant mothers during epidemics.

**Tweetable abstract:**

The COVID‐19 outbreak increased pregnant women's anxiety and affected their decision‐making.

## Tweetable abstract

The COVID‐19 outbreak increased pregnant women's anxiety and affected their decision‐making.

This article includes Author Insights, a video abstract available at https://vimeo.com/rcog/authorinsights16381

## Graphical abstract

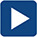

## Introduction

In late December 2019, a cluster of pneumonia caused by a novel coronavirus (2019‐nCoV) was reported.^1^ This pathogen was eventually named SARS‐CoV‐2,^2^ with its associated disease COVID‐19.^3^ By the first quarter of 2020, the COVID‐19 epidemic had become established in China. As of 3 May, a total of 82 877 cases and 4633 deaths were confirmed by the Chinese authorities.^4^ Wuhan, as the epicentre in China, accounted for 60.7% of all cases and 83.5% of deaths.^5^ Currently, SARS‐CoV‐2 has undoubtedly raised ‘a very high level of global risk’ and become the ‘public enemy number one’.^6^, ^7^ The latest data confirmed over 3 million cases and more than 0.2 million deaths outside of China.^8^ The fight against COVID‐19 has become a global priority.

The rapid transmission and life‐threatening characteristics of COVID‐19 have been reported. The public, influenced by both accurate and erroneous information, are stressed.^9^ All provinces in mainland China have adopted first‐level public health emergency (PHE) responses, including travel bans and executive orders on daily life.^10^ Consequently, the Chinese New Year holiday was disrupted and public anxiety was further aggravated.

Pregnant women, as a vulnerable population,^11^ may be of a particular concern, as anxiety has been described as a common psychological problem during pregnancy.^12^ Recent discussions of pregnancies during the COVID‐19 outbreak have mainly focused on the therapeutic aspects;^13^, ^14^, ^15^ little is known regarding mental status and psychological needs.

Prenatal care is vital to a healthy pregnancy.^16^ The emergency traffic bans have made some medical resources inaccessible and may deter women from attending routine prenatal care.^17^ Obstetricians have observed a dramatic decline in prenatal care attendance and births, as well as an increase in the caesarean section rate, all of which could threaten pregnancy outcomes. Relevant risks include ectopic pregnancy, delayed detection of fetal congenital anomalies, uncontrolled hypertension and pre‐eclampsia, post‐term delivery and dystocia.^18^, ^19^, ^20^ These adverse events may have significant consequences, possibly greater than the COVID‐19 infection itself.^9^, ^21^, ^22^ Greater science preparedness for pregnant women during public health emergencies has been advocated^23^ but we know little about women's decisions during the COVID‐19 and other PHEs.

We conducted a survey among pregnant women in Wuhan (the hardest‐hit area) and Chongqing (a neighbouring city) during the COVID‐19 outbreak, to investigate anxiety status and its influencing factors, to determine and explain key prenatal decisions and, finally, to guide social and medical practice.

## Methods

### Study design and participants

**Figure 1:**
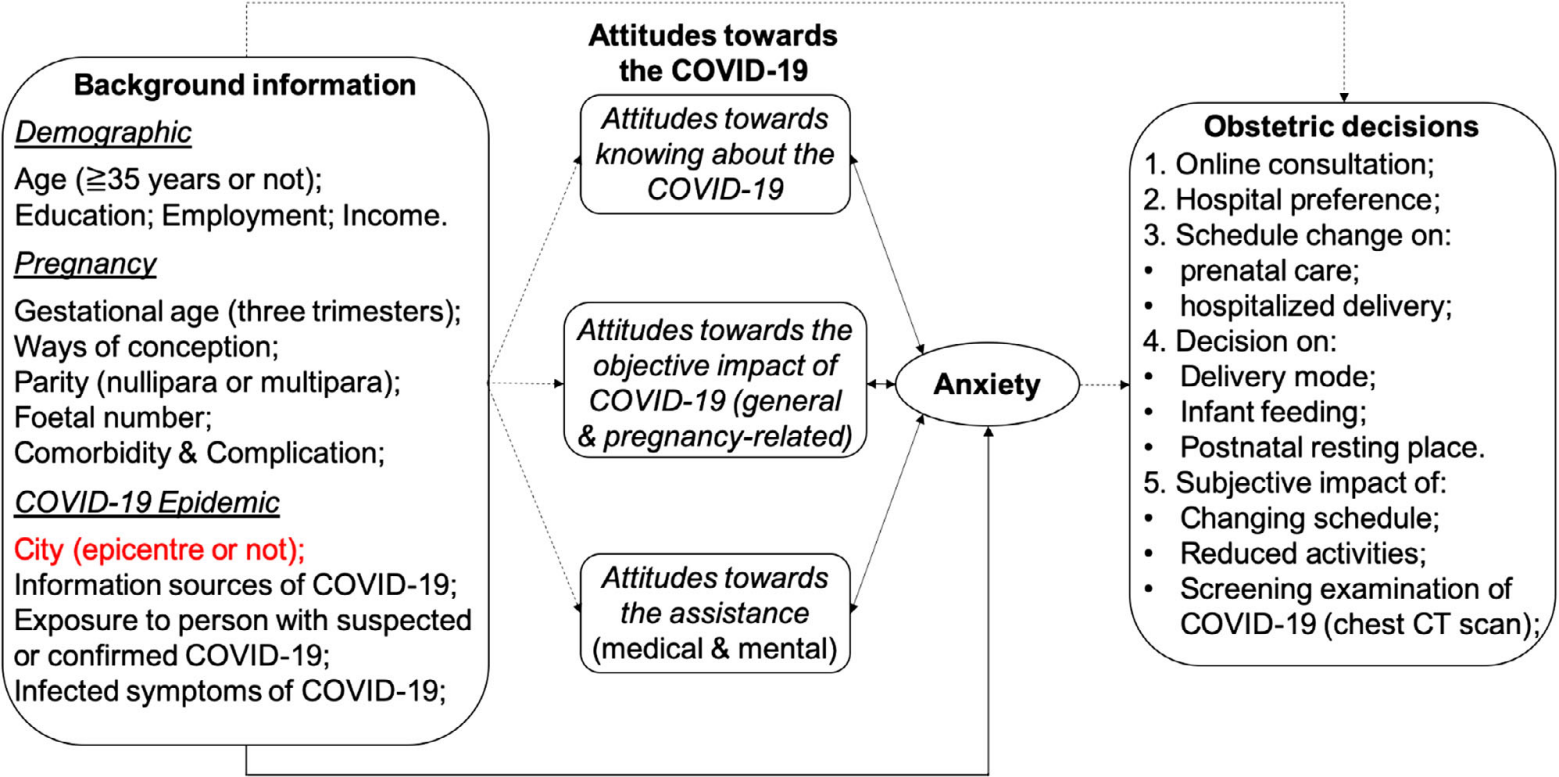
The main content of the questionnaire and the hypothesis of our study.

This is a cross‐sectional study through a self‐administered questionnaire. The anonymous survey questionnaire was designed with four modules to collect data regarding: (1) background demographic, pregnancy and COVID‐19 status; (2) attitudes towards COVID‐19; (3) anxiety status using the Self‐Rating Anxiety Scale (SAS),^24^, ^25^ which is widely used and which has been demonstrated to have excellent reliability and validity in pregnancy;^26^, ^27^ (4) obstetric decisions, defined as those pertaining to important obstetric procedures. The content of the questionnaire was reviewed and pretested by professors in Psychiatry (XYZ and his colleagues) and Obstetrics (HBQ and XL). Eventually, the validity was established following appropriate revisions. The main content of the questionnaire is shown in Figure 1 and the English version of the full questionnaire is detailed in Appendix S1.

The questionnaires were distributed from 3 to 9 February 2020, mainly through a widely used, large data platform for pregnant mothers (YunYiTong,^28^ covering more than 250 000 WeChat users nationwide), to those registered for prenatal care in hospitals in Wuhan and Chongqing. Two distribution strategies, namely, an individual WeChat message and advertising on the official accounts, were used. Additional questionnaires were distributed by obstetricians in both cities to those referred for outpatient services. Of note, a participant could only fill in this questionnaire once and only completed questionnaires could be submitted.

We followed relevant guidelines to ensure that the study was voluntary and confidential. The study was approved by the ethics committee of the First Affiliated Hospital of Chongqing Medical University, and an electronic informed consent was obtained before completing the questionnaire. The participants were not involved in the development of our study and none of the established core outcome sets has been used.

### Procedures

#### Data cleaning

**Figure 2:**
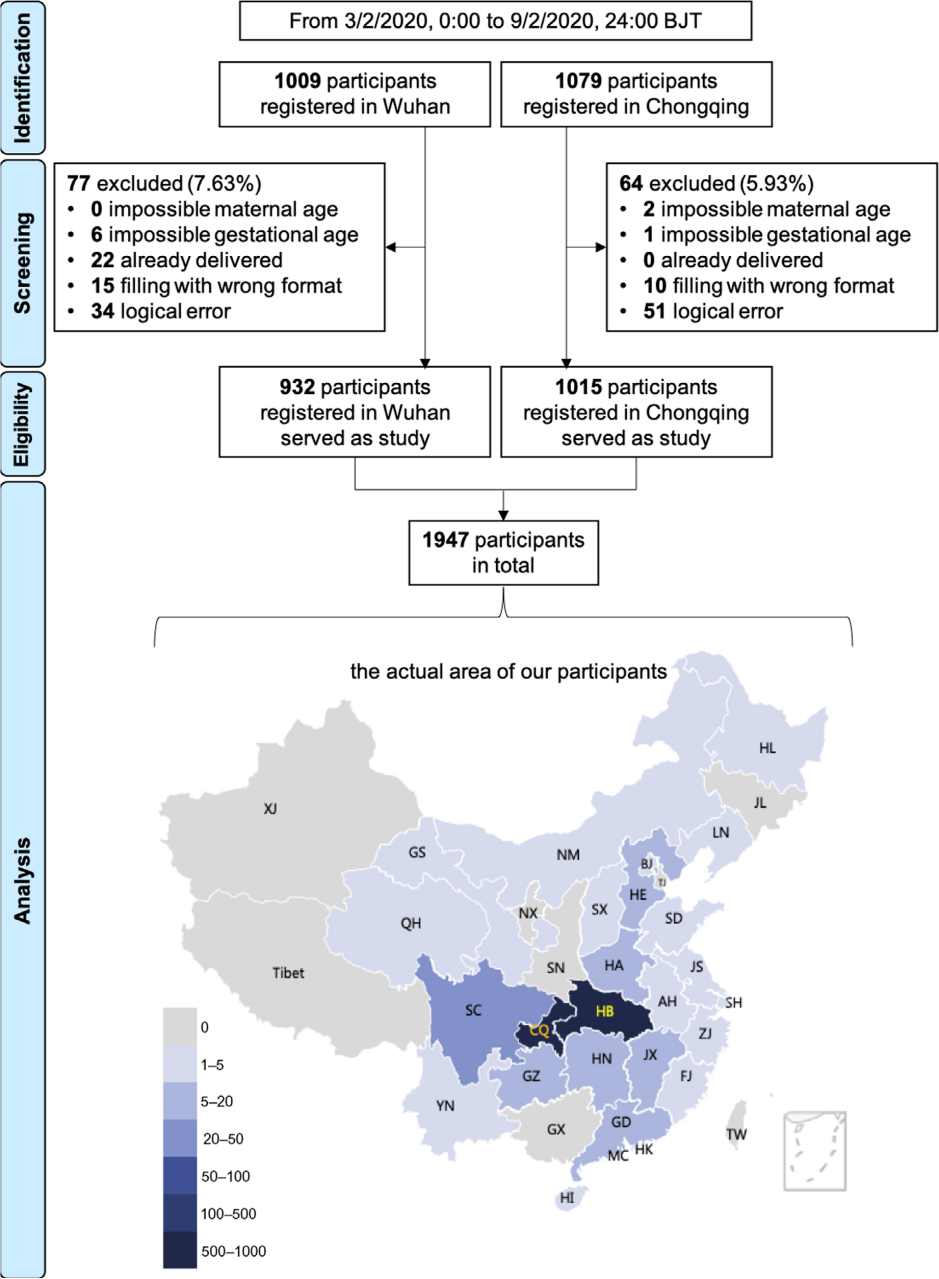
The flow chart of our study. CQ, Chongqing, an adjacent province‐level municipality of Hubei province; HB, Hubei province, the capital city of which is Wuhan.

Data collection and input were automatically conducted. All data from the questionnaires were reviewed and the following subjects were excluded: (1) maternal age <14 or >60 years; (2) non‐pregnant, with the answer of ‘already delivered’ or ‘<0 or >45 weeks of gestational age’; (3) answers with wrong format; (4) illogical answers (choosing two options that contradicted each other in multiple‐choice questions). Figure 2 shows the flow chart of our study.

#### Background information

The residency was based on both the city that the subjects registered for check‐up (Wuhan or Chongqing) and the region in which subjects actually resided at the time of survey. As a result of the Chinese Festival travel rush, these two addresses were not always the same. The registration area was used as the residency for the following analyses. As a reference, a map of the actual location of the participants is displayed in Figure 2.

We classified those aged ≥35 years as elderly gravida. Participants were assigned to one of three gestational age (GA) groups: (1) the first trimester: GA <14 complete weeks, (2) the second trimester: GA 14– 27^+6^ weeks, (3) the third trimester: GA ≥28 weeks. Parity was divided into nullipara and multipara. Other grouping standards are in accordance with the categorical options in the questionnaire (Appendix S1).

#### Assessment of attitudes towards COVID‐19

Items measuring attitudes towards COVID‐19 were designed on a five‐point scale from ‘totally disagree’ to ‘strongly agree’. Although this assessment was comprised of three sections (knowing about the COVID‐19, the objective impact of COVID‐19 and assistance given), these 11 questions were analysed separately (Appendix S1).

#### Assessment of anxiety status

The anxiety status was assessed using the Chinese version of the SAS,^25^ and the responses to the scale were summed as a standard score and a degree of anxiety by an established method:^26^, ^27^ the scores from 20 items were calculated to obtain a raw score ranging from 20 to 80, and the standard score was calculated using the raw score multiplied by 1.25. A standard score ≥50 indicates anxiety status: standard scores of 50–59, 60–69 and ≥70 were considered mild, moderate and severe anxiety, respectively.

#### Assessment of obstetric decisions

The obstetric decisions pertained to: (1) online consultation; (2) hospital preference; (3) schedule on prenatal visit or delivery; (4) the mode of delivery, infant feeding and postnatal resting; (5) the five‐point subjective impact on pregnancy of the items including changing schedule, reduced activities, and possible screening examination of COVID‐19 (e.g. chest CT scan). These unstructured questions were analysed individually (Figure 1, Appendix S1).

### Statistical analysis

We calculated the exact numbers and proportions for all variables in Wuhan and Chongqing, as well as the total for the two cities. Cronbach's alpha was used to assess the reliability of the anxiety scale. To compare the distribution of background, attitude, anxiety and obstetric decisions between the two cities, the Chi‐square test, Kruskal–Wallis test and Student's *t* test were used in accordance with the type of data.

All factors related to pregnant women's background and their attitude towards COVID‐19 were selected as independent variables. We used stepwise logistic regression models to estimate the effect of these factors on the anxiety status.

The Statistics Analysis Software version 9.4 (SAS Institute Inc., Cary, NC, USA) was used and a significance level set at *P* < 0.05 was applied. Figures presented were plotted with PRISM version 8.0 for windows (GraphPad Software Inc., San Diego, CA, USA).

The corresponding authors had full access to all of the data in the study and had the final responsibility to submit the article for publication.

## Results

A total of 1947 valid questionnaires were received, 932 from Wuhan and 1015 from Chongqing. Of these women, 866 (92.9%) and 934 (92.0%) in Wuhan and in Chongqing, respectively, stayed where they were registered during the study period (Figure 2).

### Background status

Participants reported diverse demographic, pregnancy and epidemic characteristics (Table S1). First, the general characteristics of their demographic background were of families with a middle‐level income and a working pregnant mother, although distribution differences existed between cities. Secondly, most pregnant women surveyed were in their second (32.8%) or third trimester (62.9%). There were more third‐trimester women in Wuhan (79.8 versus 47.4%). The majority of participants in both cities were nullipara and had experienced spontaneous singleton conception without comorbidity or complication, though Chongqing had a higher proportion of multiple pregnancies (4.4 versus 1.6%). Detailed distribution of comorbidity and complication is shown in Table S2. Third, information on COVID‐19 from official media were widely accepted during this period in both cities. The proportions of self‐reported symptoms were statistically the same in both cities, but the exposure history to diagnosed or suspected cases was more prevalent in Wuhan (4.7 versus 0.1%).

### Attitudes towards COVID‐19

**Figure 3:**
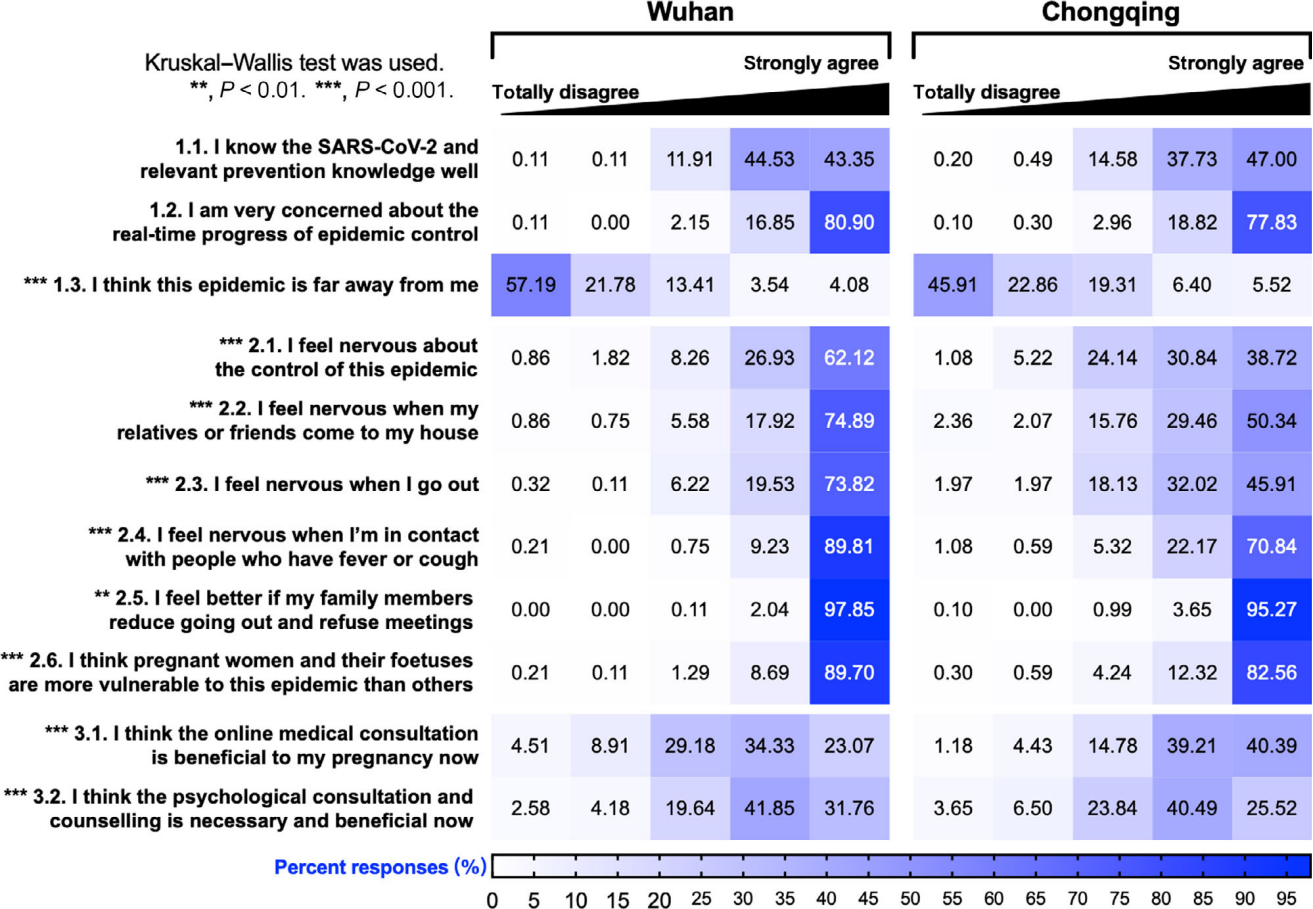
Participants' attitudes towards COVID‐19.

Attitudes towards COVID‐19 were more extreme in Wuhan, with statistically more responses of ‘totally disagree’ or ‘strongly agree’ (Figure 3). Overall, four‐fifths of mothers felt nervous about the objective impacts of COVID‐19, such as epidemic control, outdoor activity and person‐to‐person contact. Over 90% of the participants considered themselves vulnerable to this epidemic. Women in both cities held comparatively positive attitudes towards online medical consultation and psychological counselling, but differences still existed between cities (*P* < 0.001).

### Prenatal anxiety and associated factors

Cronbach's alpha for the SAS was indicative of moderate‐to‐good internal reliability: 0.78. Specific details are shown in Figure S1.

As shown in Table 1, the participants in Wuhan had significantly higher anxiety scores. The mean standard score for anxiety was 43.97 (SD 8.71) for pregnant mothers in Wuhan, with a quarter of them scoring 50 or more. In Chongqing, an average score of 40.37 (SD 7.15) was reported, among whom about 90% scored lower than 50. The overall prevalence of anxiety during this period was 17.2%. Pregnant women in the epidemic hardest‐hit area (Wuhan) were much more anxious, 18.8 and 5.7% of whom underwent mild, and moderate/severe anxiety; the corresponding proportions were 9.4 and 1.1% in Chongqing, respectively. The effect of the different cities on the SAS standard score was small to medium (effect size [ES]: Cohen's *d* 0.44).

**Table 1:** Participants' anxiety status

	City		Total (*n* = 1947)	*t*/χ^2^	*P*‐value
	Wuhan (*n* = 932)	Chongqing (*n* = 1015)			
**Standard score** *	43.97 ± 8.71	40.37 ± 7.15	42.09 ± 8.13**	9.9150	<0.0001
	42.50 (11.25)	40.00 (10.00)	41.25 (10.00)		
**Degree of anxiety** ***					
No	704 (75.54)	909 (89.56)	1613 (82.85)	69.9681	<0.0001
Mild	175 (18.78)	95 (9.36)	270 (13.87)		
Moderate or severe	53 (5.69)	11 (1.08)	64 (3.29)		

Comparations were conducted across the two cities.

a Data are mean ± SD or median (IQR). Student's *t*‐test was used.

b The Cohen's *d* for the SAS standard score was indicative of small‐to‐medium effect: 0.44.

c Data are *n* (%). Kruskal–Wallis test was used.

The multivariable analysis (Table 2) showed the impact of each of the selected background or attitude factors on anxiety when controlling for all other factors. First, pregnant women from middle‐level income families were about half as likely to report anxiety than were those earning an extremely high or low wage. Second, women in Wuhan were about twice as likely to develop anxiety (odds ratio [OR] 1.83, 95% CI 1.38–2.41). Third, those who had fever, cough, diarrhoea or symptoms of suspected infection were five times as likely to have anxiety than were otherwise healthy women (OR 4.92, 95% CI 1.84–13.17).

**Table 2:** Multivariable analysis of factors associated with anxiety

Factor type	Influence factor	*P*‐value	OR (95% CI)
Background	**Monthly household income, CNY**		
	<5000	0.0238	1 (reference)
	5000–9999	0.0336	0.6570 (0.4459–0.9680)
	10 000–49 999	0.0027	0.5477 (0.3698–0.8113)
	50 000 or more	0.5673	0.8038 (0.3804–1.6985)
Background	**City (Chongqing as reference)**	<0.0001	1.8263 (1.3814–2.4145)
Background	**Infected symptoms (No symptom as reference)**	0.0015	4.9194 (1.8370–13.1737)
Attitude*	**I know the SARS‐CoV‐2 and relevant prevention knowledge well**	0.0001	0.7114 (0.5988–0.8453)
Attitude*	**I think this epidemic is a long distance from me**	0.0312	1.1268 (1.0108–1.2560)
Attitude*	**I feel nervous about the control of this epidemic**	0.0222	1.2277 (1.0297–1.4637)
Attitude*	**I feel nervous when I go out**	0.0061	1.3359 (1.0860–1.6434)
Attitude*	**I think the online medical consultation is beneficial to my pregnancy now**	<0.0001	0.6451 (0.5704–0.7296)
Attitude*	**I think the psychological consultation and counselling is necessary and beneficial now**	0.0002	1.3145 (1.1396–1.5163)

CI, confidence interval; CNY, Chinese yuan; OR, odds ratio.

a The attitudes ranged from ‘totally disagree’ to ‘strongly agree’.

Furthermore, the attitudes towards COVID‐19 were associated with anxiety status. Those with relatively more knowledge about COVID‐19 and with a rational risk perception (not too nervous about epidemic control or going out), were less likely to be anxious. Additionally, positive attitudes towards online medical consultation demonstrated a protective feature from anxiety (OR 0.65, 95% CI 0.57–0.73), whereas those who opted for psychological consultation showed the opposite effect (OR 1.31, 95% CI 1.14–1.52).

### Obstetric decisions

The participants' obstetric decisions are summarised in Table 3.

**Table 3:** Participants' obstetric decisions

	City		Total	χ^2^	*P*‐value
	Wuhan	Chongqing			
**Request of online consultation** *	*n* = 932	*n* = 1015	*n* = 1947	8.6528	0.0033
Yes	703 (75.43)	705 (69.46)	1408 (72.32)		
No	229 (24.57)	310 (30.54)	539 (27.68)		
**Hospital preference** **	*n* = 932	*n* = 1015	*n* = 1947	–	–
Refuse to go to any hospital	390 (41.85)	281 (27.68)	671 (34.46)	43.1368	<0.0001
Previous hospital of prenatal care	319 (34.23)	713 (70.25)	1032 (53.00)	253.0501	<0.0001
Hospital closer to home	147 (15.77)	122 (12.02)	269 (13.82)	5.7467	0.0165
Large comprehensive hospital	36 (3.86)	107 (10.54)	143 (7.34)	31.8512	<0.0001
Specialized hospital of Obstetrics	420 (45.06)	145 (14.29)	565 (29.02)	223.4554	<0.0001
**Prenatal care (plan, reason/way)** **	*n* = 499	*n* = 796	*n* = 1295	–	–
Postponed, inconvenience	461 (92.38)	580 (72.86)	1041 (80.39)	74.1274	<0.0001
Postponed, afraid of infected	194 (38.88)	209 (26.26)	403 (31.12)	22.7947	<0.0001
As planned (on time), online	4 (0.80)	22 (2.76)	26 (2.01)	6.0026	0.0140
As planned (on time), face‐to‐face	13 (2.61)	172 (21.61)	185 (14.29)	90.4530	<0.0001
**Hospitalised delivery (plan, reason/way)** **	*n* = 203	*n* = 87	*n* = 290	–	–
Postponed, inconvenience	96 (47.29)	27 (31.03)	123 (42.41)	6.5891	0.0103
Postponed, afraid of infected (self)	102 (50.25)	37 (42.53)	139 (47.93)	1.4534	0.2280
Postponed, afraid of infected (baby)	104 (51.23)	37 (42.53)	141 (48.62)	1.8464	0.1742
Ahead of time, waiting for labour	35 (17.24)	9 (10.34)	44 (15.17)	2.2506	0.1336
Ahead of time, caesarean in advance	31 (15.27)	6 (6.90)	37 (12.76)	3.8370	0.0501
As planned (on time)	39 (19.21)	34 (39.08)	73 (25.17)	12.7634	0.0004
**Delivery mode** *	*n* = 932	*n* = 1015	*n* = 1947	43.0645	<0.0001
Always CS	174 (18.67)	266 (26.21)	440 (22.60)		
Always VD	588 (63.09)	657 (64.73)	1245 (63.94)		
Change from CS to VD	52 (5.58)	31 (3.05)	83 (4.26)		
Change from VD to CS	118 (12.66)	61 (6.01)	179 (9.19)		
**Infant feeding** *	*n* = 932	*n* = 1015	*n* = 1947	38.4869	<0.0001
Always breastfeeding	777 (83.37)	919 (90.54)	1696 (87.11)		
Always bottle feeding	23 (2.47)	35 (3.45)	58 (2.98)		
Change from breast to bottle	92 (9.87)	48 (4.73)	140 (7.19)		
Change from bottle to breast	40 (4.29)	13 (1.28)	53 (2.72)		
**Postnatal resting place** *	*n* = 932	*n* = 1015	*n* = 1947	71.1758	<0.0001
Always home	687 (73.71)	847 (83.45)	1534 (78.79)		
Always PSI	59 (6.33)	94 (9.26)	153 (7.86)		
Change from home to PSI	24 (2.58)	5 (0.49)	29 (1.49)		
Change from PSI to home	162 (17.38)	69 (6.80)	231 (11.86)		
**Impact of changing schedule** ***	*n* = 932	*n* = 1015	*n* = 1947	191.4008	<0.0001
Completely no impact	11 (1.18)	12 (1.18)	23 (1.18)		
Almost no impact	25 (2.68)	81 (7.98)	106 (5.44)		
Slight impact	149 (15.99)	335 (33.00)	484 (24.86)		
Comparative impact	378 (40.56)	446 (43.94)	824 (42.32)		
Significant impact	369 (39.59)	141 (13.89)	510 (26.19)		
**Impact of reduced activities** ***	*n* = 932	*n* = 1015	*n* = 1947	113.0946	<0.0001
Completely no impact	19 (2.04)	24 (2.36)	43 (2.21)		
Almost no impact	50 (5.36)	110 (10.84)	160 (8.22)		
Slight impact	194 (20.82)	332 (32.71)	526 (27.02)		
Comparative impact	371 (39.81)	425 (41.87)	796 (40.88)		
Significant impact	298 (31.97)	124 (12.22)	422 (21.67)		
**Impact of chest CT scan** ***	*n* = 932	*n* = 1015	*n* = 1947	5.5295	0.0187
Completely no impact	9 (0.97)	1 (0.10)	10 (0.51)		
Almost no impact	20 (2.15)	14 (1.38)	34 (1.75)		
Slight impact	126 (13.52)	99 (9.75)	225 (11.56)		
Comparative impact	326 (34.98)	377 (37.14)	703 (36.11)		
Significant impact	451 (48.39)	524 (51.63)	975 (50.08)		

CS, caesarean section delivery; PSI, postnatal resting institution; VD, vaginal delivery.

Data are *n* (%). Comparations were conducted across the two cities.

a Chi‐square test was used.

b Multiple choice, Chi‐square test was used for each choice.

c Kruskal–Wallis test was used.

Online consultation was requested by more than 70% of the participants, with a higher proportion in Wuhan (75.4 versus 69.5%). Absolute differences could be found between the two cities in hospital preference during this period. Of pregnant women in Wuhan, 41.9% reported refusal to go to any hospital recently, compared with 27.7% in Chongqing. Questionnaire responses revealed a general trust in previous (53.0%) and specialised (29.0%) hospitals among mothers, although differences existed in the proportion of that trust between cities (*P* < 0.0001).

Inconvenience caused by traffic bans raised significant concerns and, as a result, 80.4 and 42.4% of the 1947 participants in Wuhan and Chongqing, respectively, would defer their appointments for prenatal care and hospitalised delivery. These responses were more common in Wuhan than in Chongqing (92.4 versus 72.9% and 47.3 versus 31.0%, respectively). Fear of infection was another reason for delaying plans. With respect to prenatal care, a minority (16.3% in general) reported an ‘as planned’ visit. Very few mothers in Wuhan chose to complete their scheduled check online (*n* = 4) or face‐to‐face (2.6%). In Chongqing, however, 2.8 and 21.6% were willing to undertake their prenatal checks on time via the Internet and face‐to‐face. When it came to hospitalised delivery, 27.9% of all participants chose ‘ahead of time’; 15.2% wanted to be hospitalised earlier to wait for the onset of labour and 12.8% wanted to have a caesarean in advance. Only 25.2% of all women reported an ‘as planned’ hospitalised delivery; this proportion was higher in Chongqing (39.1 versus 19.2%).

Of pregnant women in Wuhan 12.7% would change delivery mode from vaginal delivery to caesarean section consequent upon the epidemic, whereas this proportion in Chongqing was halved (6.0%). However, the reverse change, from caesarean to vaginal delivery, was smaller (Wuhan 5.6%, Chongqing 3.1%). These city‐based differences and uneven changes in mode could also be seen in choices of infant feeding and postnatal resting. Overall, there were more women who preferred caesarean section, bottle feeding and postnatal rest at home during this period than before, especially in Wuhan.

Over 90% of pregnant women believed that there had been negative impact on their pregnancy through changing schedules and reduced activities; these subjective impacts were deemed to be more significant in Wuhan (*P* < 0.0001). Slightly more than half of the women (50.1%) thought the chest CT scan would significantly influence their pregnancy; this was more marked in Chongqing (51.6 versus 48.4%, *P* = 0.0187).

## Discussion

### Main findings

We report the first large cross‐sectional study of pregnant women's anxiety status and obstetric decision‐making during the outbreak of COVID‐19. We focused on the epicentre (Wuhan) and a neighbouring city (Chongqing) and involved 932 and 1015 participants, respectively.

#### Prenatal anxiety and associated factors

The global estimated prevalence of prenatal anxiety fluctuates between 14 and 24%.^29^, ^30^, ^31^ Unfortunately, during the study period, the overall anxiety rate was 17.2%, with a higher rate in Wuhan (24.5%) than in Chongqing (10.4%). Such a difference was probably attributed to the higher exposure and the stricter restrictions in Wuhan.

We found that women with medium‐level incomes were protected from anxiety compared with those with high‐ or low‐level wages. No other demographic characteristics or factors related to pregnancy were found to be related to anxiety, although previous studies have suggested that age, education, occupation, parity and gestational age could influence anxiety.^31^, ^32^, ^33^

Living in the epidemic centre and suffering subjective symptoms had major impacts on anxiety levels. We noted the high proportion of women who obtained their information through official media channels during the outbreak (84.3%), though the multivariable analysis did not show any association of information source with anxiety. There was higher frequency of exposure to COVID‐19 in the epicentre (Wuhan), although there were no differences in symptoms across cities (Table S1). However, symptoms, not exposure, were an independent influencing factor of anxiety.

Important attitudes towards COVID‐19, which were easy to manipulate psychologically (discussed below), were found to be associated with anxiety.

Our univariable analysis identified other potential factors that were correlated with anxiety (Table S3). We believe that the mechanism of epidemic on anxiety is unique, so regional differences greatly mask the effect of these conventional factors, which further reminds us to pay attention to prenatal anxiety during a PHE.

#### Obstetric decisions

Similar to anxiety, obstetric decisions revealed city‐based differences. Key choices changed, including the time of prenatal care or delivery, mode of delivery and infant feeding. These choices were translated into eventual outcomes, according to our clinical observations (Table S5).

Although the factors that influence obstetric decisions are difficult to determine fully, we conjecture that the severity of the epidemic is likely to be the dominant factor, although requests for online consultation and the subjective impact of chest CT scans were sensitive to gestational age (Tables 3 and S4).

Our hypothesis (Figure 1) is supported by evidence that anxiety can change prenatal decisions.^17^, ^34^ A mediation analysis should be conducted better to understand the pathway of prenatal anxiety to obstetric decisions during a PHE.

### Strengths and limitations

Wuhan as the epidemic centre was included in our study. Chongqing, a nearby city, is a good comparison, not only because of its epidemic condition (less affected but still in the outbreak; Figure S2) but also because of the relatively high accessibility of data and the demographic comparability. Nevertheless, some limitations should be considered. First, the study design suggests the possibility of self‐report bias. Second, bias might exist, as data from uncompleted questionnaires were inaccessible. Third, the baseline was not completely balanced across cities. Efforts have been made to minimise biases: multiple centres were involved, the two cities provided a large and similar sample size, and sensitivity analysis was performed.

Our participants in Wuhan and Chongqing, generated relatively reliable and representative samples. More importantly, we hope that the real data from these two cities can serve as a reference and be promoted to more regions, especially to emerging epidemic areas worldwide. Appropriate adjustments are needed before cross‐region application. In the comparative results, Chongqing was hit harder than other regions farther away from Wuhan, which may weaken the impact gap of COVID‐19. Fortunately, descriptive parameters were recorded in each city and are likely to be accessible in cities that are the subjects of future studies.

Anxiety levels may have been underestimated. Pregnant women who are not registered on the platform or the hospital did not participate and may have a lower socio‐economic status and higher anxiety levels.^33^ The inclusion of only completed questionnaires may also have led to an underestimation. In addition, the majority of pregnant women were in mid‐ or late trimester, whereas the highest level of anxiety reported previously was in early pregnancy.^33^

This was a cross‐sectional study and long‐term cohort studies on post‐traumatic stress disorder or postpartum mental state are merited.

### Interpretation

During an unpredictable period, such as a PHE, comprehensive recommendations on prenatal care are needed. Our findings can serve as a reference in the following aspects.

#### Psychological intervention

Prenatal anxiety, which may affect pregnancy outcomes,^21^, ^22^, ^35^, ^36^ should be considered carefully. Psychological intervention and corresponding public health measures during PHEs are necessary and require multidisciplinary cooperation.^9^ These interventions could be: (1) to encourage the information from authoritative sources; (2) to understand correctly the susceptibility to disease; (3) to perceive the risk rationally (avoid exaggerated perception of symptoms); (4) to use online consultation and counselling.

#### Obstetric assistance

To prevent irreversible adverse events, deferral of prenatal visits or delivery should not be standard care.^37^ Our findings also suggested that caesarean delivery and bottle feeding may have increased as women sought to avoid vertical transmission. For researchers, as new evidence comes to light,^14^, ^15^, ^38^ such transmission is likely to be rejected. Still, lessons could be learnt from similar PHEs (SARS and MERS).^39^, ^40^, ^41^ For obstetricians, the contingency plans should include the capacity to increase caesarean deliveries and manage potential perinatal infections; these plans need to be developed and implemented quickly.^42^ Importantly, authoritative information on delivery and infant feeding should be available to prenatal mothers. The education of mothers concerning radiography during pregnancy should focus on its comparative safety and diagnostic necessity in order to decrease subjects' concerns.

#### Source of support

In our study, more than 70% of participants requested online support. Digital health merits increased investment.

The restrictive measures imposed, as a consequence of the crisis, have dual effects during PHEs, and a balance between epidemic control and mental pressure is required, taking into consideration the availability of medical resources.^12^, ^31^, ^43^ Meanwhile, the rationale of opening ‘green channel’ for pregnant women has merit, as does support for this special group in the quarantine zones.

## Conclusion

The outbreak aggravated prenatal anxiety, and the associated factors could be targets for psychological attention. In parallel, key obstetric decision‐making changed, emphasising the need for pertinent professional advice. Special support is essential for pregnant mothers during epidemics.

### Disclosure of interests

None declared. Completed disclosure of interest forms are available to view online as supporting information.

### Contribution to authorship

HBQ, XL and XYL contributed to the protocol design. MMC and GQS collected data. LS and YW analysed data. JZ, YS, HZ and PNB contributed to the interpretation of results. XYL drafted the manuscript and JHW proofread the manuscript. PNB also assisted with detailed editing of the manuscript for appropriate language. HBQ and XL revised the final version and are guarantors of this manuscript. All authors made substantial contributions to the paper and read and approved the final manuscript.

### Details of ethics approval

The study was approved by the ethics committee of the First Affiliated Hospital of Chongqing Medical University on 14 February 2020 (ID:20200501). An electronic informed consent was obtained before completing the questionnaire.

### Funding

This work was supported by grants from National Natural Science Foundation of China (No. 81771614 and No. 81771613), and the National Key Research and Development Program of China (No. 2016YFC1000407). The funders had no involvement in the study design, data collection and analysis, interpretation of data or preparation of the manuscript.

### Data sharing

The data used and analysed during the current study are available from the corresponding author on reasonable request.

## Supporting information


**Figure S1.** Self‐Rating Anxiety Scale (SAS).

**Figure S2.** The stage of the epidemic in each of the two cities.

**Table S1.** Participants' background of demographic, pregnancy and COVID‐19 epidemic.

**Table S2.** Comorbidity and complication of participants.

**Table S3.** Univariable analysis of background factors associated with anxiety.

**Table S4.** Participants' obstetric decisions (on the third trimester only).

**Table S5.** Current obstetric situations in two hospitals.

**Appendix S1.** The questionnaire.

**Video S1.** Author insights.

Supplementary Material

Supplementary Material

Supplementary Material

Supplementary Material

Supplementary Material

Supplementary Material

Supplementary Material

Supplementary Material

Supplementary Material

Supplementary Material

Supplementary Material

Supplementary Material
