# Supplementary material for: Prenatal anxiety and obstetric decisions among pregnant women in Wuhan and Chongqing during the COVID‐19 outbreak: a cross‐sectional study
Source: BJOG. 2020 Aug 2;127(10):1229–40. doi: 10.1111/1471-0528.16381 (PMC7362035; doi:10.1111/1471-0528.16381)
Supplement: Supplementary file 1 — Figure S1. Self‐Rating Anxiety Scale (SAS). [file BJO-127-1229-s020.pdf]

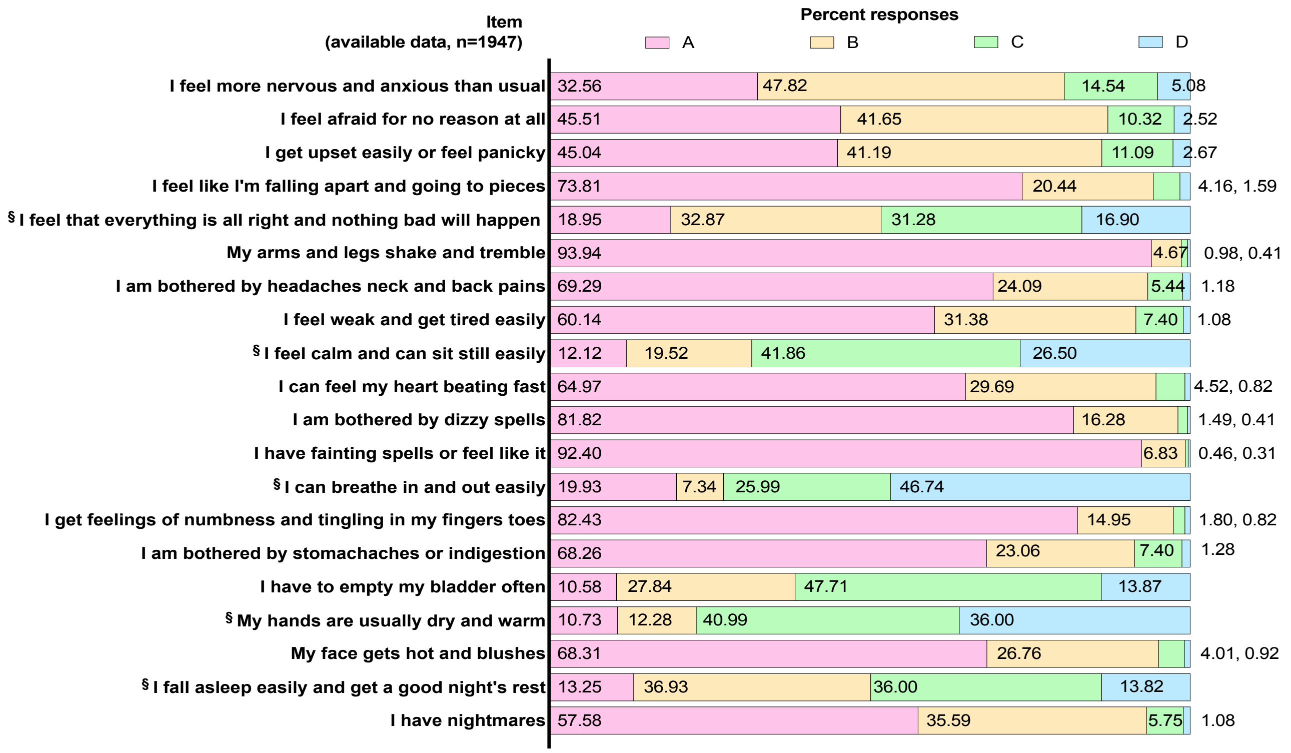

Figure S1. Self-Rating Anxiety Scale (SAS).

Alpha coefficient for the SAS was 0.78.

§, the 5 items (i.e., 5, 9, 13, 17, 19) stated positively.

A, None or a little of the time (In the past week, there have been such cases no more than one day.)

B, Some of the time (In the past week, there have been such cases in 1-2 days.)

C, Good part of the time (In the past week, there have been such cases in 3-4 days.)

D, Most or all of the time (In the past week, there have been such cases in 5-7 days.)
