# Supplementary material for: Prenatal anxiety and obstetric decisions among pregnant women in Wuhan and Chongqing during the COVID‐19 outbreak: a cross‐sectional study
Source: BJOG. 2020 Aug 2;127(10):1229–40. doi: 10.1111/1471-0528.16381 (PMC7362035; doi:10.1111/1471-0528.16381)
Supplement: Supplementary file 2 — Figure S2. The stage of the epidemic in each of the two cities. [file BJO-127-1229-s019.pdf]

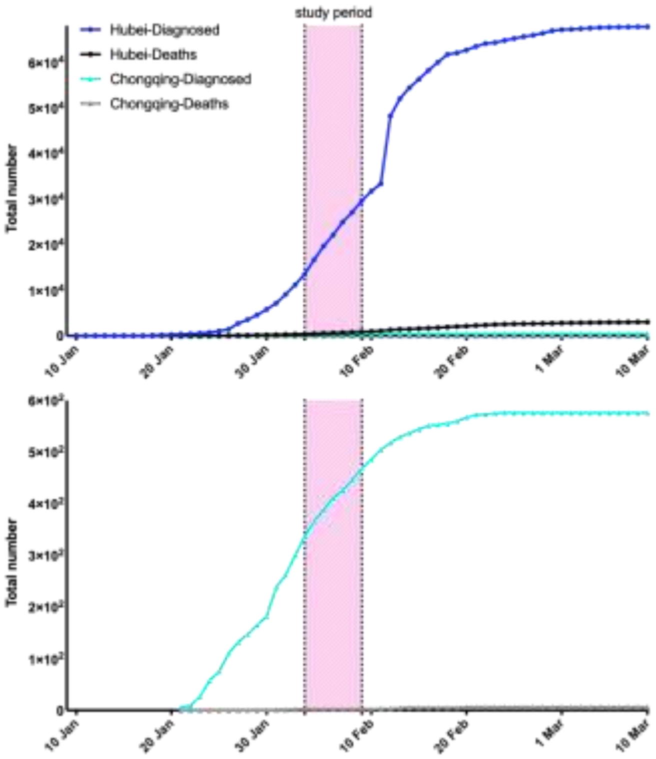


**Figure S2.** The stage of the epidemic in each of the two cities.

Authoritative data concerning the epidemic situation above come from National Health Commission of the People's Republic of China (http://www.nhc.gov.cn/, accessed 23 Apr, 2020) and Health Commission of Chongqing (http://wsjkw.cq.gov.cn/, accessed 23 Apr, 2020).
