# Supplementary material for: Prenatal anxiety and obstetric decisions among pregnant women in Wuhan and Chongqing during the COVID‐19 outbreak: a cross‐sectional study
Source: BJOG. 2020 Aug 2;127(10):1229–40. doi: 10.1111/1471-0528.16381 (PMC7362035; doi:10.1111/1471-0528.16381)
Supplement: Supplementary file 3 — Table S1. Participants' background of demographic, pregnancy and COVID‐19 epidemic. [file BJO-127-1229-s018.pdf]

Table S1. Participants' background of demographic, pregnancy and COVID-19 epidemic

	City		Total (n=1947)	χ2	*P-value*
	**Wuhan** (n=932)	**Chongqing** (n=1015)			
**Maternal age, years**			/	3.5481	0.0596
<35	843(90.45)	891(87.78)	1734(89.06)		
≧35	89(9.55)	124(12.22)	213(10.94)		
**Highest educational background**			/	1.3353	0.5129
Junior middle school and below	31(3.33)	26(2.56)	57(2.93)		
Senior middle school	106(11.37)	125(12.32)	231(11.86)		
University and above	795(85.30)	864(85.12)	1659(85.21)		
**Employment status**			/	29.0612	<0.0001
No	302(32.40)	219(21.58)	521(26.76)		
Yes	630(67.60)	796(78.42)	1426(73.24)		
**Monthly household income, CNY**			/	7.9822	0.0464
< 5,000	100(10.73)	108(10.64)	208(10.68)		
5,000-9,999	389(41.74)	440(43.35)	829(42.58)		
10,000-49,999	407(43.67)	449(44.24)	856(43.97)		
50,000 or more	36(3.86)	18(1.77)	54(2.77)		
**Gestational age, trimester**			/	241.8110	<0.0001
First	42(4.51)	41(4.04)	83(4.26)		
Second	146(15.67)	493(48.57)	639(32.82)		
Third	744(79.83)	481(47.39)	1225(62.92)		
**Ways of conception**			/	1.5030	0.2202
Spontaneous pregnancy	857(91.95)	948(93.40)	1805(92.71)		
Assisted reproductive technology	75(8.05)	67(6.60)	142(7.29)		
**Parity**			/	0.5229	0.4696
Nullipara	674(72.32)	719(70.84)	1393(71.55)		
Multipara	258(27.68)	296(29.16)	554(28.45)		
**Foetal number**			/	12.9739	0.0003
One (singleton)	917(98.39)	970(95.57)	1887(96.92)		
Two or more (multiple)	15(1.61)	45(4.43)	60(3.08)		
**Comorbidity and Complication** [A]			/	0.8242	0.3639
No	843(90.45)	930(91.63)	1773(91.06)		
Yes	89(9.55)	85(8.37)	174(8.94)		
**Information sources of COVID-19**			/	3.7567	0.1528
Official media	771(82.73)	870(85.71)	1641(84.28)		
Unofficial media	150(16.09)	132(13.00)	282(14.48)		
Other	11(1.18)	13(1.28)	24(1.24)		
**Exposure history to COVID-19**			/	45.9812	<0.0001
No	888(95.28)	1014(99.90)	1902(97.69)		
Yes	44(4.72)	1(0.10)	45(2.31)		
**Infected symptoms of COVID-19**			/	1.7974	0.1800
No	920(98.71)	1008(99.31)	1928(99.02)		
Yes	12(1.29)	7(0.69)	19(0.98)		

Data are n (%). Comparisons were conducted across the two cities. Chi-squared test was used.
CNY, Chinese yuan. A, Comorbidity and Complication (see **Table S2**).
