# Supplementary material for: Prenatal anxiety and obstetric decisions among pregnant women in Wuhan and Chongqing during the COVID‐19 outbreak: a cross‐sectional study
Source: BJOG. 2020 Aug 2;127(10):1229–40. doi: 10.1111/1471-0528.16381 (PMC7362035; doi:10.1111/1471-0528.16381)
Supplement: Supplementary file 4 — Table S2. Comorbidity and complication of participants. [file BJO-127-1229-s002.pdf]

Table S2. Comorbidity and complication of participants

	City		Total
	**Wuhan** (n=89)	**Chongqing** (n=85)	(n=174)
Threatened miscarriage or preterm labour	16(17.98)	13(15.29)	29(16.67)
Foetal complication	12(13.48)	6(7.06)	18(10.34)
Placenta abnormality	5(5.62)	8(9.41)	13(7.47)
Hypertensive disorder	13(14.61)	8(9.41)	21(12.07)
Gestational diabetes mellitus	63(70.79)	46(54.12)	109(62.64)
Intrahepatic cholestasis	1(1.12)	4(4.71)	5(2.87)
Thyroid diseases (hypothyroidism)	6(6.74)	8(9.41)	14(8.05)
Other system diseases	3(3.37)	4(4.71)	7(4.02)

Data are n (%).

This is a multiple-choice question. We made reclassifications based on the answers we obtained.
