# Supplementary material for: Prenatal anxiety and obstetric decisions among pregnant women in Wuhan and Chongqing during the COVID‐19 outbreak: a cross‐sectional study
Source: BJOG. 2020 Aug 2;127(10):1229–40. doi: 10.1111/1471-0528.16381 (PMC7362035; doi:10.1111/1471-0528.16381)
Supplement: Supplementary file 5 — Table S3. Univariable analysis of background factors associated with anxiety. [file BJO-127-1229-s001.pdf]

Table S3. Univariable analysis of background factors associated with anxiety

	Anxiety			χ2	*P-value*
	No	Mild	Moderate / Severe		
**Employment status**				5.1763	0.0229
No	416(79.85)	78(14.97)	27(5.18)		
Yes	1197(83.94)	192(13.46)	37(2.59)		
**Gestational age, trimester**				9.9127	0.0070
First	73(87.95)	10(12.05)	0(0)		
Second	550(86.07)	71(11.11)	18(2.82)		
Third	990(80.82)	189(15.43)	46(3.76)		
**Comorbidity and Complication**				4.9146	0.0266
No	1479(83.42)	240(13.54)	54(3.05)		
Yes	134(77.01)	30(17.24)	10(5.75)		
**Information sources of COVID-19**				7.5997	0.0224
Official media	1375(83.79)	217(13.22)	49(2.99)		
Unofficial media	221(78.37)	47(16.67)	14(4.96)		
Other	17(70.83)	6(25.00)	1(4.17)		
**Exposure history to COVID-19**				9.2994	0.0023
No	1583(83.23)	260(13.67)	59(3.10)		
Yes	30(66.67)	10(22.22)	5(11.11)		

(With statistical difference, but not including those identified by multivariable analysis)
Data are n (%). Kruskal-Wallis test was used.
