# Supplementary material for: Prenatal anxiety and obstetric decisions among pregnant women in Wuhan and Chongqing during the COVID‐19 outbreak: a cross‐sectional study
Source: BJOG. 2020 Aug 2;127(10):1229–40. doi: 10.1111/1471-0528.16381 (PMC7362035; doi:10.1111/1471-0528.16381)
Supplement: Supplementary file 6 — Table S4. Participants' obstetric decisions (on the third trimester only). [file BJO-127-1229-s021.pdf]

**Table S4.** Participants' obstetric decisions (on the third trimester only)

| | City | | Total | χ2 | *P-value* |
|---|---|---|---|---|---|
| | **Wuhan** | **Chongqing** | | | |
| **Request of online consultation** [A] | (n=744) | (n=481) | (n=1225) | 0.1209 | 0.7281 |
| Yes | 571(76.75) | 365(75.88) | 936(76.41) | | |
| No | 173(23.25) | 116(24.12) | 289(23.59) | | |
| **Hospital preference** [B] | (n=744) | (n=481) | (n=1225) | / | / |
| Refuse to go to any hospital | 299(40.19) | 102(21.21) | 401(32.73) | 47.8060 | <0.0001 |
| Previous hospital of prenatal care | 258(34.68) | 369(76.72) | 627(51.18) | 206.6162 | <0.0001 |
| Hospital closer to home | 114(15.32) | 59(12.27) | 173(14.12) | 2.2503 | 0.1336 |
| Large comprehensive hospital | 26(3.49) | 40(8.32) | 66(5.39) | 13.3220 | 0.0003 |
| Specialized hospital of Obstetrics | 348(46.77) | 66(13.72) | 414(33.80) | 142.6425 | <0.001 |
| **Prenatal care (plan, reason/way)** [B] | (n=391) | (n=356) | (n=747) | / | / |
| Postponed, inconvenience | 365(93.35) | 279(78.37) | 644(86.21) | 35.1742 | <0.001 |
| Postponed, afraid of infected | 156(39.90) | 107(30.06) | 263(35.21) | 7.9117 | 0.0049 |
| As planned (on time), online | 3(0.77) | 13(3.65) | 16(2.14) | 7.3965 | 0.0065 |
| As planned (on time), face-to-face | 7(1.79) | 53(14.89) | 60(8.03) | 43.2720 | <0.001 |
| **Hospitalized delivery (plan, reason/way)** [B] | (n=201) | (n=86) | (n=287) | / | / |
| Postponed, inconvenience | 95(47.26) | 27(31.40) | 122(42.51) | 6.2058 | 0.0127 |
| Postponed, afraid of infected (self) | 102(50.75) | 36(41.86) | 138(48.08) | 1.9050 | 0.1675 |
| Postponed, afraid of infected (baby) | 104(51.74) | 37(43.02) | 141(49.13) | 1.8316 | 0.1759 |
| Ahead of time, waiting for labour | 35(17.41) | 9(10.47) | 44(15.33) | 2.2398 | 0.1345 |
| Ahead of time, caesarean in advance | 31(15.42) | 6(6.98) | 37(12.89) | 3.8261 | 0.0505 |
| As planned (on time) | 38(18.91) | 34(39.53) | 72(25.09) | 13.6389 | 0.0002 |
| **Delivery mode** [A] | (n=744) | (n=481) | (n=1225) | 10.8341 | 0.0127 |
| Always CS | 146(19.62) | 113(23.49) | 259(21.14) | | |
| Always VD | 457(61.42) | 309(64.24) | 766(62.53) | | |
| Change from CS to VD | 41(5.51) | 20(4.16) | 61(4.98) | | |
| Change from VD to CS | 100(13.44) | 39(8.11) | 139(11.35) | | |
| **Infant feeding** [A] | (n=744) | (n=481) | (n=1225) | 15.0342 | 0.0018 |
| Always breast feeding | 627(84.27) | 437(90.85) | 1064(86.86) | | |
| Always bottle feeding | 17(2.28) | 13(2.70) | 30(2.45) | | |
| Change from breast to bottle | 74(9.95) | 23(4.78) | 97(7.92) | | |
| Change from bottle to breast | 26(3.49) | 8(1.66) | 34(2.76) | | |
| **Postnatal resting place** [A] | (n=744) | (n=481) | (n=1225) | 31.0503 | <0.0001 |
| Always home | 553(74.33) | 400(83.16) | 953(77.80) | | |
| Always PSI | 42(5.56) | 40(8.32) | 82(6.69) | | |
| Change from home to PSI | 12(1.61) | 4(0.83) | 16(1.31) | | |
| Change from PSI to home | 137(18.41) | 37(7.69) | 174(14.20) | | |
| **Impact of changing schedule** [C] | (n=744) | (n=481) | (n=1225) | 109.7851 | <0.0001 |
| Completely no impact | 8(1.08) | 5(1.04) | 13(1.06) | | |
| Almost no impact | 17(2.28) | 27(5.61) | 44(3.59) | | |
| Slight impact | 100(13.44) | 142(29.52) | 242(19.76) | | |
| Comparative impact | 295(39.65) | 227(47.19) | 522(42.61) | | |
| Significant impact | 324(43.55) | 80(16.63) | 404(32.98) | | |
| **Impact of reduced activities** [C] | (n=744) | (n=481) | (n=1225) | 47.7869 | <0.0001 |
| Completely no impact | 10(1.34) | 9(1.87) | 19(1.55) | | |
| Almost no impact | 36(4.84) | 42(8.73) | 78(6.37) | | |
| Slight impact | 136(18.28) | 123(25.57) | 259(21.14) | | |
| Comparative impact | 305(40.99) | 230(47.82) | 535(43.67) | | |
| Significant impact | 257(34.54) | 77(16.01) | 334(27.27) | | |
| **Impact of chest CT scan** [C] | (n=744) | (n=481) | (n=1225) | 2.3083 | 0.1287 |
| Completely no impact | 7(0.94) | 1(0.21) | 8(0.65) | | |
| Almost no impact | 17(2.28) | 6(1.25) | 23(1.88) | | |
| Slight impact | 101(13.58) | 48(9.98) | 149(12.16) | | |
| Comparative impact | 262(35.22) | 185(38.46) | 447(36.49) | | |
| Significant impact | 357(47.98) | 241(50.10) | 598(48.82) | | |

Data are n (%). Comparations were conducted across the two cities.
A, Chi-squared test was used.
B, multiple choice, Chi-squared test was used for each choice.
C, Kruskal-Wallis test was used.
CS, caesarean section delivery. VD, vaginal delivery. PSI, postnatal resting institution.
