# Supplementary material for: Prenatal anxiety and obstetric decisions among pregnant women in Wuhan and Chongqing during the COVID‐19 outbreak: a cross‐sectional study
Source: BJOG. 2020 Aug 2;127(10):1229–40. doi: 10.1111/1471-0528.16381 (PMC7362035; doi:10.1111/1471-0528.16381)
Supplement: Supplementary file 7 — Table S5. Current obstetric situations in two hospitals. [file BJO-127-1229-s003.pdf]

Table S5. Current obstetric situations in two hospitals

	Wuhan [A]			Chongqing [B]		
	**Feb, 2019**	**Feb, 2020**	**Change**	**Feb, 2019**	**Feb, 2020**	**change**
ANC	27,254	5,410	↓80.15%	16,120	6,859	↓57.45%
Births	2,038	2,090	↑2.55%	662	497	↓24.92%
CS rates	46.96%	47.42%	↑0.46%	55.33%	60.51%	↑5.18%

A, Data from Maternal and Child Health Hospital of Hubei Province.

B, Data from the First Affiliated Hospital of Chongqing Medical University.

ANC, antenatal care or prenatal care. CS, caesarean section.
