# Supplementary material for: Prenatal anxiety and obstetric decisions among pregnant women in Wuhan and Chongqing during the COVID‐19 outbreak: a cross‐sectional study
Source: BJOG. 2020 Aug 2;127(10):1229–40. doi: 10.1111/1471-0528.16381 (PMC7362035; doi:10.1111/1471-0528.16381)
Supplement: Supplementary file 8 — Appendix S1. The questionnaire. [file BJO-127-1229-s004.pdf]

Appendix S1. The questionnaire.

Notice:

- **This is the translated version (from Chinese to English) of the questionnaire.**
- **This is merely the main content of the questionnaire, not covering the informed consent, the brief introduction, the tips for filling in and the acknowledgments.**
- **Unless otherwise mentioned, the questions are single-choice questions with options below or blank-filling questions with a horizontal line.**
- **Bolded fonts were barely used to sort questions and cannot been seen by participants.**

Part One: Background Information

1. Demographic:

1.1. **Age:** Your age is ______

1.2. **Education:** Your highest educational background

A: junior middle school and below

B: senior middle school (including technical secondary school)

C: university and above (including junior college)

1.3. **Employment:** Your employment status

A: no, unemployed B: yes, currently working

1.4. **Income:** Family monthly income (including you and your spouse)

A: < 5,000 CNY B: 5,000-9,999 CNY

C: 10,000-49,999 CNY D: >50,000 CNY

2. Pregnant:

2.1. **Gestational age:** Your gestational age: _____week(s) + _____day(s)

2.2. **Ways of conception:** How did you get pregnant

A: spontaneous pregnancy

B: by assisted reproductive technology (IVF-ET)

2.3. **Parity:** How many children do you have (not including this pregnancy):______

2.4. **Foetal number:** Number of foetus in this pregnancy

A: one (singleton) B: two or more (multiple)

2.5. **Comorbidity and Complication:**

(1) Do you have any pregnancy comorbidity or complication?

A: no B: yes

If "yes", automatically switch to (2), otherwise participants do not need to complete this.

(2) What are these (multiple choices allowed)?

A: threatened miscarriage: vaginal bleeding or prevention of miscarriage

B: threatened preterm labour

C: foetal malformation or suspected malformation: chromosomal or structural abnormalities

D: foetal growth restriction (FGR)

E: foetal distress or suspected distress: possible hypoxia or low volume of amniotic fluid

F: placenta previa (PP)

G: hypertensive disorder complicating pregnancy

H: gestational diabetes mellitus (GDM)

I: intrahepatic cholestasis of pregnancy (ICP)

J: other comorbidity or complication______

3. Epidemic:

3.1. City: Wuhan or Chongqing, from backstage (the source of the questionnaire)

3.2. **Information sources:** What is your main information source regarding COVID-19?

A: official media (TV news, government official accounts, hospital official account, etc.)

B: unofficial media (friends, family members, unofficial accounts, etc.)

C: other (community campaign, etc.)

3.3. **Exposure:**

Are there any COVID-19 diagnosed or suspected case among your family or friends?

A: no B: yes

3.4. **Infected symptoms:**

Do you or your family members have any symptom of fever, cough, diarrheal, etc.?

A: no B: yes

Part Two: Attitudes

There are 12 items, please read each item carefully.

There are five options after each item, which respectively indicates:

A. totally disagree B. comparative disagree

C. neutral attitude D. comparative agree E. strongly agree

1. Attitudes towards knowing about the COVID-19

1.1. I know the SARS-CoV-2 and relevant prevention knowledge well.

1.2. I am very concerned about the real-time progress of epidemic control.

1.3. I think this epidemic is far away from me.

2. Attitudes towards the objective impact of COVID-19

2.1. **(general)** I feel nervous about the control of this epidemic.

2.2. **(general)** I feel nervous when my relatives or friends come to my house.

2.3. **(general)** I feel nervous when I go out.

2.4. **(general)** I feel nervous when I'm in contact with people who have fever or cough.

2.5. **(general)** I feel better if my family members reduce going out and refuse meetings.

2.6. **(pregnancy-related)**

I think pregnant women and their foetuses are more vulnerable to this epidemic than others.

3. Attitudes towards the assistance

3.1. **(medical)** I think the online medical consultation is beneficial to my pregnancy now.

3.2. **(mental)** I think the psychological consultation and counselling is necessary and beneficial now.

Part Three: Anxiety

There are 20 items, please read each item carefully.

There are four choices after each item, which respectively indicates:

A: None or a little of the time (In the past week, there have been such cases no more than one day.)

B: Some of the time (In the past week, there have been such cases in 1-2 days.)

C: Good part of the time (In the past week, there have been such cases in 3-4 days.)

D: Most or all of the time (In the past week, there have been such cases in 5-7 days.)
1. I feel more nervous and anxious than usual
2. I feel afraid for no reason at all
3. I get upset easily or feel panicky
4. I feel like I'm falling apart and going to pieces
5. I feel that everything is all right and nothing bad will happen
6. My arms and legs shake and tremble
7. I am bothered by headaches, neck and back pains
8. I feel weak and get tired easily
9. I feel calm and can sit still easily
10. I can feel my heart beating fast
11. I am bothered by dizzy spells
12. I have fainting spells or feel like it
13. I can breathe in and out easily
14. I get feelings of numbness and tingling in my fingers, toes
15. I am bothered by stomach-aches or indigestion
16. I have to empty my bladder often
17. My hands are usually dry and warm
18. My face gets hot and blushes
19. I fall asleep easily and get a good night's rest
20. I have nightmares

Part Four: Obstetric Decisions

1. **Request of online consultation:** Are you willing to consult doctors online?
   A: yes B: no

2. **Hospital preference:** Recently (within 1 month), your choice of hospital for prenatal check or delivery is mainly based on (multiple choices allowed)
   A: Refuse to go to any hospital.
   B: Go to the hospital where the previous routine check was conducted.
   C: Go to a hospital closer to my home.
   D: Go to a large comprehensive hospital.
   E: Go to a specialized hospital of Obstetrics.

3. **Schedule change:** According to your original plan, do you plan to see a doctor in the near future (within 1 month) for prenatal care or hospitalized delivery
   A: Yes, there is an appointment for prenatal check in the near future.
   B: Yes, there is an appointment for hospitalized delivery in the near future.
   C: No, there is no plan for prenatal check or hospitalized delivery in the near future.

If "A" in question "3", automatically switch to "3.1"

3.1. Do you think your recent prenatal care has been affected by this epidemic? (multiple choices allowed)

A: Yes, it will be postponed, because of the traffic bans and the inconvenience of attending hospital.

B: Yes, it will be postponed, because of the worries about myself being infected in hospital.

C: No, it will be done as planned, because online remote medical check-up is available recently.

D: No, it will be done as planned. I will take protective measures and see my doctor face-to-face.

If "B" in question "3", automatically switch to "3.2"

3.2. Do you think your recent hospitalized delivery has been affected by this epidemic? (multiple choices allowed)

A: Yes, it will be postponed, because of the traffic bans and the inconvenience of attending hospital.

B: Yes, it will be postponed, because of the worries about myself being infected in hospital.

C: Yes, it will be postponed, because of the worries about my new-born baby being infected.

D: Yes, I will consider hospitalization ahead of time to wait for delivery, because of the traffic bans and the inconvenience of attending hospital.

E: Yes, I will consider hospitalization ahead of time to have a caesarean section in advance, because of the worries about the uncertain influence of this epidemic whilst I am waiting at home.

F: No, it will be done as planned. I will take protective measures and go to hospital on time.

Otherwise, participants do not need to answer these two questions above.

4. Decisions:

4.1. Whether your choice of delivery mode changes due to the epidemic

A: No, I'm always going to prefer vaginal delivery if possible.

B: No, I'm always going to prefer caesarean delivery.

C: Yes, I planned vaginal delivery but the epidemic changed my mind to caesarean delivery.

D: Yes, I planned caesarean delivery but the epidemic changed my mind to vaginal delivery.

4.2. Whether your choice of lactation mode changes due to the epidemic

A: No, I always prefer breastfeeding if possible.

B: No, I always prefer bottle feeding.

C: Yes, I planned breastfeeding but the epidemic changed my mind to bottle feeding.

D: Yes, I planned bottle feeding but the epidemic changed my mind to breastfeeding.

4.3. Whether your choice of postnatal resting place changes due to the epidemic

A: No, I always intend to be at home.

B: No, I always intend to go to a specialized postnatal resting institution.

C: Yes, I changed my mind from home to a specialized postnatal resting institution.

D: Yes, I changed my mind from the postnatal resting institution to stay at home.

5. **Subjective impact:**

There are 3 items, please read each item carefully.

There are five options after each item, which respectively indicates:

A: completely no impact on my pregnancy
B: almost no impact on my pregnancy

C: slight impact on my pregnancy
D: comparative impact on my pregnancy

E: significant impact on my pregnancy

5.1. The schedule change of prenatal check or delivery due to the epidemic has…

5.2. Reduced activities due to the requirement of epidemic control has…

5.3. Contingency plan of COVID-19 screening examination (chest CT scan) has…
