# Supplementary material for: Prenatal anxiety and obstetric decisions among pregnant women in Wuhan and Chongqing during the COVID‐19 outbreak: a cross‐sectional study
Source: BJOG. 2020 Aug 2;127(10):1229–40. doi: 10.1111/1471-0528.16381 (PMC7362035; doi:10.1111/1471-0528.16381)
Supplement: Supplementary file 9 — Video S1. Author insights. [file BJO-127-1229-s005.docx]

Edited video abstract has been uploaded to the Google Drive: <https://drive.google.com/file/d/1kOAXdkMZTbeoBZDUV21o5TbkPWL6M9h6/view?usp=sharing>
